# Frailty and comorbidity in COVID-19 patients with and without ICU admission restrictions: a retrospective observational study

**DOI:** 10.1186/s44158-025-00268-5

**Published:** 2025-08-08

**Authors:** Felix Starlander, Erland Östberg

**Affiliations:** 1 Department of Anaesthesia and Intensive Care, Västmanland Hospital Västerås, Västerås, Sweden; 2 Centre for Clinical Research Västmanland, Uppsala University, Uppsala, Sweden

**Keywords:** COVID-19, ICU triage, Clinical Frailty Scale, Charlson Comorbidity Index, Resource allocation

## Abstract

**Background:**

Limited resources during the COVID-19 pandemic made ICU admission decisions ethically complex. In Sweden, where ICU bed availability per capita is among the lowest in Europe, clinical judgment guided triage decisions, as prognostic scoring systems like the Clinical Frailty Scale (CFS) and Age-adjusted Charlson Comorbidity Index (ACCI) were not routinely used. This study aimed to compare patients considered eligible for intensive care with those for whom ICU admission was restricted and to evaluate whether a post hoc assessment of frailty and comorbidity aligned with clinical decision making.

**Methods:**

This retrospective observational study included 204 COVID-19-positive patients admitted to a Swedish secondary hospital during the first pandemic wave. Patients were categorized as either eligible for intensive care (ICU group) or having a documented ICU admission restriction (ICU restriction group). Electronic medical records were reviewed to assign CFS and ACCI scores, and a combined score was calculated to better reflect overall frailty and comorbidity burden.

**Results:**

The ICU group had a mean age of 68 years versus 83 years in the ICU restriction group. Of the ICU group, 26 out of 100 patients (26%) were ultimately admitted to intensive care. Median combined CFS + ACCI scores were 5 (IQR 5–6) in the ICU group and 12 (IQR 11–14) in the ICU restriction group; difference in score: 7 (95% CI, 6–8; *p* < 0.001). The combined score demonstrated clear separation between the groups with minimal overlap: in the ICU group, 95% of patients had a combined score below 8.3, while in the ICU restriction group, 95% of patients had a score above 8.4.

**Conclusions:**

Marked contrasts in age, frailty, and comorbidity burden distinguished patients eligible for intensive care from those with an ICU admission restriction, reflecting a close correspondence between the prognostic scoring systems and clinical judgment. Integrating the CFS and ACCI into a single combined score sharpened the analysis and may prove useful in future research or as a triage tool, although further validation of this approach is warranted.

## Introduction

The COVID-19 pandemic led to concerns in many countries regarding potential lack of intensive care resources. It was evident that elderly and frail patients [1] as well as patients with hypertension, diabetes, and obesity were more severely affected by the disease [2, 3]. In many countries, the virus was heavily transmitted throughout residential facilities and homes for the aged during the initial phase. The complexity of a novel disease with absence of long-term survival data, its impact on the elderly population and patients with certain risk factors, together with limited intensive care resources, made ethical decision-making exceptionally challenging. In Sweden, where the number of intensive care beds per capita is among the lowest in Europe [4], extensive discussions were held on decision-making regarding referrals and admissions to intensive care. The Swedish National Board of Health and Welfare published a national guideline regarding restrained intensive care resources and how to prioritize during “extraordinary circumstances” [5]. In essence, patients who had the greatest probability of long-term survival were to be prioritized. Although this may seem intuitive, frontline physicians experienced significant ethical pressure when determining which patients should receive intensive care.

In other parts of the world, the Clinical Frailty Scale (CFS) was used as an aid to decide whether a patient was a candidate for intensive care or not [6, 7]. In addition, the Age-adjusted Charlson Comorbidity Index (ACCI) [8, 9] was suggested as a predictive nomogram for triaging hospitalized patients with COVID-19 [10]. However, none of these prognostic scoring tools were used routinely in Sweden during the initial phase of the pandemic.

Västerås Hospital is a public secondary hospital with one of the lowest intensive care capacities per capita in the country. The characteristics and outcomes of COVID-19 patients who received intensive care at this hospital during the first phase of the pandemic have been previously reported [11]. The aim of this study was to compare frailty and comorbidities between admitted patients considered eligible for intensive care and those for whom an early decision was made to limit care, restricting admission to the intensive care unit (ICU). This comparison also aimed to assess the concordance between clinical judgment and two established scoring tools, the CFS and the ACCI, in identifying patients with respiratory failure who may benefit from intensive care.

## Methods

This retrospective observational study was approved by the Swedish Ethical Review Authority (approval number 2020-03372) with a waiver of informed consent. It was conducted in accordance with the 1996 Declaration of Helsinki and reported in accordance with the Strengthening the Reporting of Observational Studies in Epidemiology (STROBE) statement [12].

### Setting

Västerås Hospital in Region Västmanland is located west of Stockholm and provides public secondary care to a regional population of approximately 280,000 inhabitants. Pre-pandemic, the ICU had 2.9 beds/100,000 inhabitants, compared with the national average of 5.1 ICU beds/100,000 inhabitants [13], being one of the lowest in Europe [4].

The region experienced a 2-week delay in the first wave of the viral outbreak compared to Stockholm, allowing time for reorganization and logistical adjustments. One post-anaesthesia care unit was rearranged to an enclosed ICU with a capacity of 14 beds, dedicated to COVID-19 patients. Meanwhile, the general ICU capacity for non-COVID-19 patients was reduced from eight to five beds. Both ICUs were able to deliver standard intensive care, for example, invasive ventilatory support and renal replacement therapy, but not extracorporeal membrane oxygenation. Two primary medical wards at the hospital were reorganized to host non-ICU patients with COVID-19, one of which had the capacity to treat patients with high-flow nasal oxygen therapy.

In Region Västmanland, frontline physicians were increasingly encouraged to make early decisions about whether an admitted patient was eligible for intensive care or should have a care limitation restricting ICU admission. The decision also served as a triage tool to determine ward placement. As high-flow oxygen therapy was a limited resource, only patients who would be considered for intensive care in the event of clinical deterioration were admitted to the medical ward where it was available. Any patient without a documented decision to forgo intensive care could, depending on the clinical course of the disease, be considered for subsequent ICU admission.

### Study population

We included all patients from the age of 60 with a positive severe acute respiratory syndrome coronavirus 2 (SARS-CoV-2) real-time reverse transcription polymerase chain reaction (RT-PCR) admitted to Västerås Hospital between March 1 and June 30, 2020. The time interval encompassed the entire first wave of the pandemic in this region. Exclusion criteria were patients with a previously documented ICU admission restriction or do-not-resuscitate order.

### Data collection

One of the authors (FS) reviewed the electronic medical record (Cosmic R82.05, Cambio HealthCare Systems, Sweden) to retrieve patient characteristics and clinical data from the time of hospital admission, along with any relevant information documented prior to that time. Of particular interest were documented decisions regarding life-sustaining treatment, including do-not-resuscitate orders and care limitations, such as ICU admission restrictions. Patients were subsequently divided into two groups: those deemed eligible for intensive care (ICU group) and those who were restricted from ICU admission (ICU restriction group). During the study period, nearly all care-limiting decisions were made at the time of admission or within the first 24 h. A small number of patients underwent a later evaluation that ultimately resulted in a care-limiting decision restricting ICU admission; these individuals were included in the ICU restriction group.

The medical records were carefully searched for any data describing patients’ functional status before and during the hospital stay, such as Activities of Daily Living (ADL), physical ability, and level of dependency. Functional status was defined by the updated CFS, which is a pictographic tool to evaluate frailty based on functional status and activity [14, 15]. A score from 1, being the absolute fittest of their age, to 9, being terminally ill and approaching the end of life, is given.

The ACCI [8, 9] was used to quantify the additional burden of comorbidity and age. The ACCI assigns weighted points to seventeen defined medical conditions, and additional points are added for age: 1 point per decade over 50 years (Table 1). Patient’s comorbidities were recorded from either the medical record or by searching each patient’s documented list of International Classification of Diseases 10th revision (ICD-10) codes.

**Table 1:** Comorbidities included in the Age-adjusted Charlson Comorbidity Index

Comorbidity	Score
Myocardial infarction Congestive heart failure Cerebral vascular disease Peripheral vascular disease Dementia Chronic obstructive pulmonary disease Connective tissue disease Peptic ulcer disease Mild liver disease Diabetes, uncomplicated Age^a^	1
Hemiplegia Moderate/severe renal disease Diabetes with end-organ damage Any solid tumor Lymphoma Leukemia	2
Moderate/severe liver disease	3
Metastatic solid tumor Acquired immunodeficiency syndrome (AIDS)	6

^a^Age: 50–59 years: 1 point; 60–69 years: 2 points; 70–79 years: 3 points; ≥ 80 years: 4 points

To describe the combined burden of frailty and comorbidity, we merged the points from each scoring system by simple addition, with the result presented separately.

Length of stay, in-hospital mortality, mortality 30 days after discharge, and mortality 1 year after discharge were recorded. Length of stay was defined as the duration from admission to discharge or death. If readmission occurred, the total number of days in hospital was calculated. New diagnoses, comorbidities, or deterioration of functional status that became apparent during a patient’s hospital stay were not recorded or included in the analysis.

### Statistical analysis

Normally distributed quantitative data were expressed as means (standard deviations) and analyzed using Welch’s *t*-test. Non-normally distributed data were presented as medians (interquartile ranges) and analyzed using Mann–Whitney *U* test. Normality was evaluated by visual judgment of histograms. The differences between group medians regarding CFS, ACCI, and the combined scores were analyzed by Mann–Whitney *U* test and presented together with corresponding 95% confidence intervals (CI), estimated using the percentile bootstrap method with 2000 resamples, implemented via the *confintr* R package. Categorical data were reported as numbers (percentages) and analyzed using the *χ*^2^ test or Fisher’s exact test for variables with cell counts less than five in the contingency table. A two-sided *p* value of < 0.05 was considered statistically significant. Statistical analyses were done in R studio using R statistical programming language version 2024.04.2 (R Core Team, R Foundation for Statistical Computing, Vienna).

## Results

In total, 208 patients admitted to hospital fulfilled the inclusion criteria. Four of these were excluded due to a previously existing decision on limiting life-sustaining treatment. Thus, 204 patients were included in the study analysis. Insufficient documentation on functional status prevented the estimation of CFS for three patients in the ICU group and one patient in the ICU restriction group. In addition, missing information precluded the calculation of BMI for several patients. For all other variables, no data were missing.

Patient characteristics and comorbidities are presented in Table 2. The mean age in the ICU group and the ICU restriction group was 68 and 83 years, respectively. Regarding comorbidity, 40% of the patients in the ICU group had at least one comorbidity recorded other than age, compared to 91% in the ICU restriction group. All comorbidities were more common in the ICU restriction group except for uncomplicated diabetes and mild liver disease (Table 2). No patients with moderate to severe chronic kidney disease, dementia, hemiplegia, or tumors were in the ICU group. Dementia was present in 31% of the patients in the ICU restriction group.

**Table 2:** Patient characteristics and comorbidities

	ICU group (*n *= 100)	ICU restriction group (*n *= 104)	*p* value
Age, years	68 (5)	83 (8)	< 0.001
Male sex	67 (67)	62 (60)	0.274
Body Mass Index, kg/m^2^	29 (4.9)^a^	28 (4.8)^b^	0.239
Comorbidities			
Obesity	28 (28)	29 (28)	0.741
Hypertension	54 (54)	67 (65)	0.130
Diabetes, uncomplicated	13 (13)	9 (9)	0.317
Diabetes with complications	12 (12)	26 (25)	0.017
COPD or other pulmonary disease	11 (11)	20 (19)	0.102
Myocardial infarction	6 (6)	20 (19)	0.005
Congestive heart failure	4 (4)	29 (28)	< 0.001
Peripheral vascular disease	1 (1)	13 (13)	0.001
Cerebral vascular accident or TIA	4 (4)	30 (29)	< 0.001
Hemiplegia	0 (0)	10 (10)	0.002
Chronic kidney disease, moderate to severe	0 (0)	13 (13)	< 0.001
Connective tissue disease	3 (3)	7 (7)	0.332
Peptic ulcer disease	5 (5)	8 (8)	0.431
Liver disease, mild	3 (3)	2 (2)	0.679
Liver disease, moderate to severe	0 (0)	0 (0)	N/A
Dementia	0 (0)	32 (31)	< 0.001
Solid tumor, localized	0 (0)	6 (6)	0.029
Solid tumor, metastatic	0 (0)	7 (7)	0.014

Data are expressed as mean (SD) or number (%)

*COPD* chronic obstructive pulmonary disease, *TIA* transient ischemic attack

^a^*n *= 79, 21 missing values for BMI in the ICU group

^b^*n = 85*, 19 missing values for BMI in the ICU restriction group

**Fig. 1:**
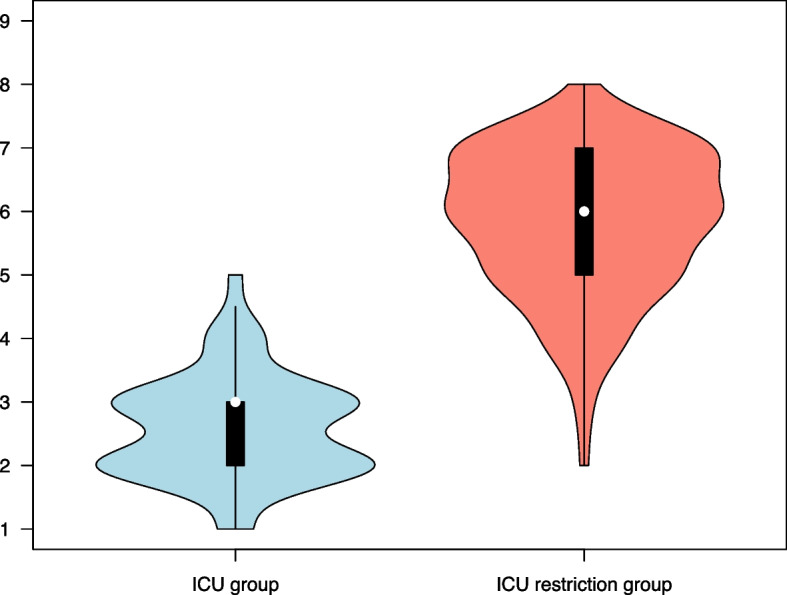
Distribution of Clinical Frailty Scale scores (1–9) in the two groups. Violin plots with overlaid box plots indicating the median (white dot), interquartile range (box), and range (whiskers), excluding outlier observations. The difference in median scores between groups was 3 (95% CI 3–4; *p* < 0.001, Mann–Whitney *U* test). ICU group, *n *= 97; ICU restriction group, *n *= 103

**Fig. 2:**
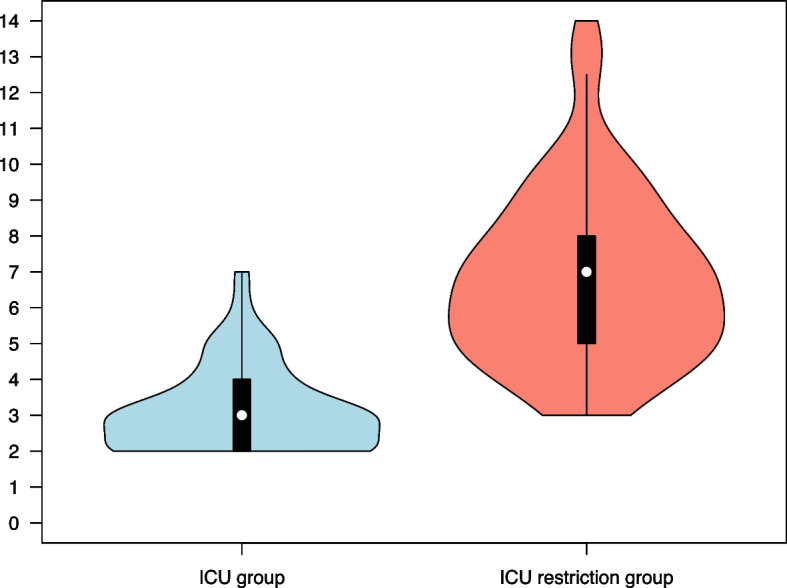
Distribution of Age-adjusted Charlson Comorbidity Index scores in the two groups. Violin plots with overlaid box plots indicating the median (white dot), interquartile range (box), and range (whiskers), excluding outlier observations. The difference in median scores between groups was 4 (95% CI 3–4; *p* < 0.001, Mann–Whitney *U* test). ICU group, *n *= 100; ICU restriction group, *n *= 104

**Fig. 3:**
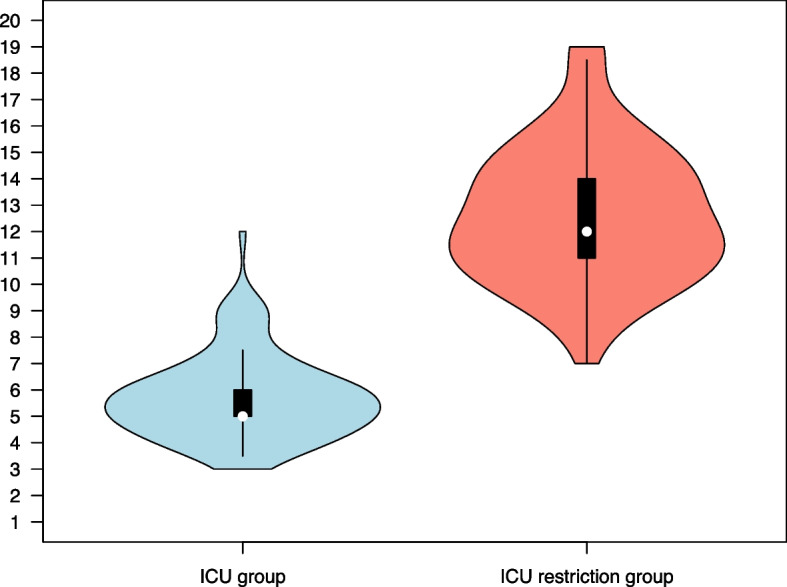
Distribution of the combined scores of Clinical Frailty Scale + Age-adjusted Charlson Comorbidity Index in the two groups. Violin plots with overlaid box plots indicating the median (white dot), interquartile range (box), and range (whiskers), excluding outlier observations. The difference in median scores between groups was 7 (95% CI 6–8; *p* < 0.001, Mann–Whitney *U* test). ICU group, *n *= 97; ICU restriction group, *n *= 103

The CFS and ACCI scores, as well as the combined score (CFS + ACCI) for the two groups are presented in Table 3. The distribution of these scores within each group is illustrated in Figs. 1, 2, and 3, respectively. In the ICU group, 95% of the patients had a combined CFS + ACCI score of less than 8.3, and in the ICU restriction group, 95% of the patients had a combined CFS + ACCI score above 8.4.

**Table 3:** Frailty and comorbidity scores, length of stay and mortality data

	ICU group (*n* = 100)	ICU restriction group (*n *= 104)	Difference (95% CI)	*p* value
Scoring system				
CFS	3 (2–3)^a^	6 (5–7)^b^	3 (3–4)	< 0.001
ACCI	3 (2–4)	7 (5–8)	4 (3–4)	< 0.001
CFS + ACCI	5 (5–6)^a^	12 (11–14)^b^	7 (6–-8)	< 0.001
Length of stay, days	7 (4–13)	9 (6–14)		0.153
Mortality				
In-hospital mortality	13 (13)	38 (37)		< 0.001
Mortality 30 days after discharge	0 (0)	5 (5)		0.060
Mortality 1 year after discharge	0 (0)	8 (8)		0.007

Data are expressed as median (IQR) or number (%)

*ACCI* Age-adjusted Charlson Comorbidity Index, *CFS *Clinical Frailty Scale, *CI *confidence interval

^a^*n *= 97, three missing values for CFS in the ICU group

^b^*n *= 103, one missing value for CFS in the ICU restriction group

In the ICU group, one patient had a notably high combined score of 12, which deviated from the rest of the group (Fig. 3). This 63-year-old patient had a relatively low CFS of 5, but several comorbidities, including chronic heart failure, previous myocardial infarction, stroke, and diabetes, that contributed additional points to the combined score. However, all comorbidities were considered mild.

Similarly, in the ICU restriction group, one patient deviated from the overall pattern with a notably low combined score of 7, overlapping with many of the scores observed in the ICU group (Fig. 3). This patient was an 85-year-old, physically active individual whose only comorbidities were hypertension and uncomplicated diabetes. In contrast, other patients in the ICU restriction group with relatively low combined scores of 8 to 9 had additional comorbidities not captured by the ACCI, such as neuromuscular disorders, aortic stenosis, or severe single-organ disease.

In-hospital mortality, as well as mortality at 30 days and 1 year after discharge, were all higher in the ICU restriction group. Among patients deemed eligible for intensive care (ICU group), 26 out of 100 (26%) were ultimately admitted to the ICU during their hospital stay [11].

## Discussion

In this retrospective observational study, marked contrasts in age, frailty, and comorbidity burden distinguished COVID-19 patients eligible for intensive care from those with an ICU admission restriction. Although a noticeable difference between the groups was anticipated, it proved to be more pronounced than expected and most clearly demonstrated when combining the CFS and the ACCI score. The strong alignment between the scoring systems and clinical judgment suggests that the CFS, ACCI, or the combined score could serve as primary screening tools to help clinicians identify patients with respiratory failure who may benefit from intensive care.

Regarding clinical frailty, most patients in the ICU group had a CFS score of 2 or 3, and none had a score higher than 5 (Fig. 1). Apparently, without having CFS incorporated as a decision tool during the pandemic, the clinicians’ judgements seemed to be in accordance with guidelines in countries where CFS at the time was recommended as a tool for triaging [6]. The difference between the two groups in median CFS score was 3 (95% CI 3–4), a difference associated with a considerable increase in hazard ratio [16]. Furthermore, in the ICU restriction group, the median CFS score of 6 (IQR 5–7) has been shown to correlate with a high degree of mortality in general [7, 17, 18], as well as in COVID-19 patients [19].

When considering specific comorbidities in our study population, the presence of dementia was the comorbidity that differed most between the two groups. Several factors may contribute to clinicians’ decisions not to admit patients with dementia to intensive care, particularly for mechanical ventilation. These include potential autonomic dysregulation, increased risk of aspiration, higher incidence of delirium, and communication difficulties that may hinder participation in physiotherapy.

The overall burden of comorbidities was assessed using the ACCI, a validated tool and strong predictor of mortality, including ICU mortality in patients with COVID-19 [20, 21]. In our study, the difference between the ICU group and the ICU restriction group in median ACCI score (4; 95% CI 3–4) is from a clinical perspective substantial. An early validation of the ACCI indicated a 1.4-fold increase in the relative risk of death for each additional comorbidity rank; however, this evaluation was conducted before the pandemic and focused on perioperative complications [9]. The ICU restriction group in our study demonstrated a high ACCI score of median 7 (IQR 5–8). Studies on COVID-19 patients have shown an in-hospital mortality rate of 18% for those with an ACCI ≤ 4, compared to 41% for those with an ACCI > 4 [22], confirming that a high ACCI is an independent predictor of mortality in this patient population. Still, ACCI has several limitations. Because the index primarily uses a dichotomous scoring system, it does not account for the severity of individual comorbidities. For example, a patient with congestive heart failure and severely reduced ejection fraction receives the same score (one point) as a patient with well-managed heart failure. Therefore, relying solely on the ACCI as a triage tool may be misleading, potentially leading to less severely ill patients being incorrectly deemed ineligible for intensive care. In addition, the ACCI does not account for several critical conditions that can significantly impact a patient's chances of surviving intensive care, for example, neuromuscular diseases, morbid obesity, and immunosuppression other than AIDS.

The shortcomings described above were also evident in our study population. Some patients were excluded from intensive care due to low functional status, despite having few comorbidities. Conversely, others had an acceptable functional status but a significant comorbidity burden which formed the basis for the decision to restrict ICU admission. This illustrates the limitations of relying on a single scoring system to assess a patient’s potential to benefit from intensive care. We believe it is appropriate to consider both frailty and existing comorbidities when making decisions about ICU admission and life-sustaining treatment, in line with previous recommendations [23, 24].

Therefore, and to refine the analysis, we combined the two scoring systems by simple addition. The resulting composite score, illustrated in Fig. 3, reflects the cumulative burden of frailty and comorbidity. In this study, it sharpened the distinction between the two groups and supports the impression that clinical decision-making remained consistent and robust, despite the extraordinary pressures of the pandemic.

In addition, the combined score facilitated the identification of two outliers, which are further described in the Results section. Nonetheless, these two individuals highlight the limitations of applying population-based screening systems at the individual level and underscore the importance of patient-centred care.

As the population continues to age, physicians will be confronted with an increasing number of challenging ethical decisions regarding intensive care triage. Importantly, this is not solely a matter of appropriately utilizing limited resources but also concerns the potential harm or suffering that patients with high risk of mortality or morbidity may endure from intensive care once it has been initiated [25]. Finally, regardless of the outcome from any scoring system or any clinical judgment, the patient’s own wishes regarding life-sustaining treatment should, whenever possible, be actively sought and considered.

## Limitations

This study has several limitations. First, although previous research has shown that CFS scores can be reliably assigned through retrospective review of medical records [26, 27], we used a single investigator to conduct both the review and the CFS assessments. While this may enhance consistency, it does not preclude the possibility of bias.

Second, we did not account for the severity of each patient’s COVID-19 illness, as we did not collect data on blood test results, oxygen saturation levels, or oxygen requirements. Nor did we take into account how far into their illness the patient was at the time of the decision.

Third, it is important to remember that patients admitted to hospital during the pandemic represented an already selected group of patients, where general practitioners and doctors working in nursing homes or sheltered accommodations made independent decisions on ceiling of care and therefore did not admit many frail patients to the hospital.

Fourth, given the retrospective design of the study, we did not apply formal corrections for multiple testing. Reported p values should therefore be interpreted as descriptive and with caution.

Fifth, although it appeared useful in this study to combine the CFS and ACCI scores to describe and compare patient groups undergoing triage for potential life-sustaining treatment, the strategy may be questioned and has not been validated.

## Conclusion

COVID-19 patients deemed eligible for intensive care and those with an ICU admission restriction were clearly distinguishable in terms of age, frailty, and comorbidity burden, reflecting strong alignment between prognostic scoring tools and clinical judgment. Combining the CFS and ACCI into a single composite score improved the analysis and may support future research or serve as a triage tool, although further validation of this approach is warranted.

## Data Availability

The datasets supporting the conclusions of this article are available from the corresponding author upon reasonable request.

## Abbreviations

COVID-19: Coronavirus disease 2019
ICU: Intensive care unit
CFS: Clinical Frailty Scale
ACCI: Age-adjusted Charlson Comorbidity Index
